# The Reading Signatures of Agreement Attraction

**DOI:** 10.1162/opmi_a_00047

**Published:** 2021-11-01

**Authors:** Sol Lago, Carlos Acuña Fariña, Enrique Meseguer

**Affiliations:** Goethe University Frankfurt; University of Santiago de Compostela; University of La Laguna

**Keywords:** agreement attraction, comprehension, cue-based retrieval, similarity-based interference, Spanish

## Abstract

The comprehension of subject-verb agreement shows “attraction effects,” which reveal that number computations can be derailed by nouns that are grammatically unlicensed to control agreement with a verb. However, previous results are mixed regarding whether attraction affects the processing of grammatical and ungrammatical sentences alike. In a large-sample eye-tracking replication of Lago et al. (2015), we support this “grammaticality asymmetry” by showing that the reading profiles associated with attraction depend on sentence grammaticality. In ungrammatical sentences, attraction affected both fixation durations and regressive eye-movements at the critical disagreeing verb. Meanwhile, both grammatical and ungrammatical sentences showed effects of the attractor noun number prior to the verb, in the first- and second-pass reading of the subject phrase. This contrast suggests that attraction effects in comprehension have at least two different sources: the first reflects verb-triggered processes that operate mainly in ungrammatical sentences. The second source reflects difficulties in the encoding of the subject phrase, which disturb comprehension in both grammatical and ungrammatical sentences.

## INTRODUCTION

Over the past 30 years agreement has been a popular topic in the literature on syntactic processing. This research started with a focus on the agreement errors made in sentence production (Bock & Miller, 1991) and later addressed how these errors are processed in comprehension (Nicol et al., 1997). The interest in agreement is well-founded because it displays many features that make language processing an exciting research area. First, agreement often capitalizes on the existence of a morphological component, whose productivity is known to differ crosslinguistically. For instance, whereas the English phrase “*all the white broken dishes*” contains one morphological plural marker, there are five in its Spanish translation (“*todos los platos blancos rotos*”). Further, agreement can occur between nonadjacent elements, thus implicating the use of working memory (Lewis & Vasishth, 2005). Finally, agreement can be studied in production (where processing goes from meaning to form) or comprehension (where form precedes meaning), and it can be evaluated with different methodologies: judgment tasks, self-paced reading, event-related potentials. Here we use the eye-tracking method to establish the reading signatures of the comprehension of agreement in Spanish, a language with previous conflicting findings.

In production, subject-verb agreement errors sometimes occur when a noun phrase contains a plural modifier: speakers are more likely to accidentally produce a plural verb in sentences like (1b) than (1a), consistent with the idea that the plural modifier “attracts” the verb, misleading agreement computations. These so-called attraction errors are crosslinguistically robust in production, having been attested in languages like English, Spanish (Vigliocco et al., 1996), Italian (Vigliocco et al., 1995), French (Franck et al., 2002), Dutch (Hartsuiker et al., 2003), and Russian (Lorimor et al., 2008), among others.

(1)  a. *The key to the **cabinet** are rusty from many years of disuse.
     b. *The key to the **cabinets** are rusty from many years of disuse.

A dominant model of these errors, known as “marking and morphing,” was originally developed as a production model (Eberhard et al., 2005). Marking and morphing maintains that attraction arises during the computation of the number of the subject phrase. The plural feature of the attractor noun *cabinets* affects this computation and renders it more likely to be plural in (1b) than in (1a). On this account, the subject phrase number is misconstrued, and the verb merely receives the result of the previous miscalculation (Bock & Cutting, 1992; Bock & Eberhard, 1993; Bock & Miller, 1991; Eberhard, 1997; Vigliocco et al., 1996; Vigliocco & Nicol, 1998). One strength of the marking and morphing model is that it can capture a commonly observed pattern known as the *number markedness effect*: number mismatch effects tend to be stronger when the head noun is singular and the attractor plural (as in 1b) than in the reversed arrangement (e.g., “*The keys to the cabinet*”). According to the model, the lack of a morphological singular marker reduces the contribution of the attractor to the subject number computation.

Meanwhile, comprehension studies are more recent and originally sought to evaluate whether the configurations responsible for attraction in production elicited processing differences in comprehension. But an important advantage of comprehension studies is that they make it possible to explicitly manipulate the number of the verb triggering an agreement computation: Whereas verb number is decided by participants in production, in comprehension researchers can manipulate whether a plural or a singular verb appears after the subject phrase. Therefore, comprehension designs include not only ungrammatical sentences such as (1a–b) but also their grammatical counterparts (2a–b), in which the auxiliary verb is singular and agrees in number with the subject head:

(2)  a. The key to the **cabinet** is rusty from many years of disuse.
     b. The key to the **cabinets** is rusty from many years of disuse.

A clear prediction of the marking and morphing model is that if attraction occurs in comprehension, it should affect both grammatical and ungrammatical sentences. This is because both versions—and thus subject number computations—are identical prior to the (un)grammatical verb. Crucially, the marking and morphing model predicts that attraction should yield opposite effects depending on grammaticality. A plural attractor should *facilitate* the processing of the ungrammatical verb: If participants are more likely to misconstrue the subject phrase as plural in (1b), they should sometimes fail to notice the agreement violation at the plural auxiliary in (1b) compared to (1a), which has an unambiguously singular subject.

By contrast, a marking and morphing account predicts that a plural attractor should *disrupt* the comprehension of a grammatical verb. If participants are more likely to misconstrue the subject phrase as plural in (2b), they should mistake the singular auxiliary for an agreement violation. This should result in more processing difficulty in (2b) than in (2a), which contains an unambiguously singular subject. In short, the marking and morphing model predicts that a plural attractor should disrupt processing in a grammatical sentence like (1b) but facilitate it in an ungrammatical sentence like (2b).

An alternative account, cue-based retrieval, makes different predictions (Engelmann et al., 2019; Jäger et al., 2017; Jäger et al., 2020; Lewis & Vasishth, 2005; for a recent overview, see Vasishth et al., 2019). While there are several computational implementations of cue-based retrieval, they all propose that attraction does not result from a faulty encoding of the subject phrase, but rather from similarity-based interference during the retrieval process initiated by a verb. When a verb is encountered, speakers use the syntactic, semantic, and morphological features of the verb as cues to retrieve an appropriate number controller from memory. Memory chunks corresponding to preceding items in the sentence are queried in parallel, and the chunk with the most features matching the cues of the verb is the most likely to be retrieved.

In an ungrammatical sentence like (1b), interference at retrieval is high if there is a plural attractor, because the subject head matches the syntactic cues of the verb but the plural attractor matches the number cue. Due to this partial match, the plural attractor is sometimes misretrieved, allowing comprehenders to license the verb in number and resulting in processing facilitation: This effect is known as *facilitatory interference*. By contrast, interference in grammatical sentences is low with a plural attractor, because the subject head fully matches the verb in syntactic and number cues while the attractor does not. By contrast, retrieval interference in grammatical sentences is higher with a singular attractor, as the number overlap between both nouns increases competition at retrieval: This effect is known as *inhibitory interference*. In short, retrieval-based accounts predict that plural attractors should facilitate processing in both grammatical and ungrammatical sentences like (1b) and (2b).

To summarize, both marking and morphing and retrieval-based accounts predict that plural attractors should ease the processing of ungrammatical verbs. However, their predictions differ for grammatical sentences. Marking and morphing predicts that plural attractors should corrupt the representation of the subject phrase and disrupt the processing of grammatical verbs. By contrast, retrieval-based accounts predict that plural attractors should reduce interference at retrieval and facilitate the processing of grammatical verbs. Thus, the study of attraction effects in grammatical and ungrammatical sentences is critical to arbitrate between different accounts of attraction. Which of them is more consistent with previous results?

### Attraction in Comprehension

Unfortunately, attraction effects in comprehension have a mixed empirical record. The text below summarizes four contested empirical patterns related to potential differences between grammatical and ungrammatical sentences. Our study was an attempt to clarify these patterns through the use of a large participant sample and a paradigm—reading eye-tracking—with good temporal resolution.

#### First Issue: The Grammaticality Asymmetry.

Early work supported the predictions of the marking and morphing account (Nicol et al., 1997; Pearlmutter et al., 1999). For instance, in two self-paced reading and one eye-tracking study, Pearlmutter and colleagues (1999) showed that a plural attractor created difficulty in grammatical sentences but eased difficulty in ungrammatical ones. These effects appeared sometimes at the verb but more often at the word following it. When ungrammatical verbs were preceded by plural attractors, participants showed shorter total reading times and fewer first-pass regressions than when verbs were preceded by singular attractors. But after grammatical verbs, the opposite was found, with longer total reading times and more regressions with plural attractors. Therefore, the authors concluded that the plural attractor occasionally caused the subject to be misrepresented as plural in both production and comprehension. In the literature since then, accounts placing the origin of attraction in the equivocal representation of the subject phrase are known as *representational accounts* (Eberhard et al., 2005; Franck et al., 2002; Hammerly et al., 2019; Hartsuiker et al., 2001; Staub, 2009).

However, while later studies largely replicated attraction effects in ungrammatical sentences, they raised questions about their existence in grammatical sentences. Wagers and colleagues (2009) were the first to reason that in grammatical sentences such as “*The key to the **cabinets** is* …” there is a danger of misattributing attraction to an unrelated factor: the difficulty of processing a plural noun per se. Plurals are morphologically and semantically more complex than singulars, and noun number influences processing times (Lau et al., 2007; Lehtonen & Laine, 2003; New et al., 2004). Therefore, processing difficulty in grammatical sentences may reflect the downstream effects of having previously encountered the plural noun “*cabinets*,” rather than attraction. This confound is mitigated in ungrammatical sentences because attraction there emerges as *faster* reading times at the verb after a plural attractor, which is the opposite than expected from a spillover effects of noun plurality.

Wagers and colleagues (2009) addressed the potential confound in two ways. First, they incorporated another word after the plural noun, in order to decrease the cost of attractor plurality prior to the verb, for example, “*The key to the cabinets unsurprisingly was/were* … .” Second, they tested for attraction using object relative clauses (RC), in which the attractor did not linearly intervene between the RC subject and the verb, for example, “*The cabinets that the key opens/open* … .” In several self-paced reading studies, they observed that attraction in grammatical sentences was absent when either an adverb was added or a non-intervening attractor was used. Importantly, attraction effects still occurred in ungrammatical sentences. Therefore, it was proposed that in comprehension attraction occurred only in ungrammatical sentences, with little or no effect in grammatical ones. This pattern is known as the “grammaticality asymmetry.” In the factorial 2 × 2 designs often used in comprehension (resulting in the four conditions 1a–b and 2a–b above), the asymmetry is operationalized as an interaction between the factors grammaticality and attractor number, which demonstrates quantitatively different attraction effects in grammatical vs. ungrammatical sentences.

Currently, the empirical status of the grammaticality asymmetry is unclear, as shown by a recent review by Hammerly et al. (2019). On the one hand, studies that have measured number attraction effects in both grammatical and ungrammatical sentences have indeed found significant effects more often in ungrammatical sentences: 91% vs. 38%, respectively. However, only 60% of the 45 studies that tested the grammaticality asymmetry found a significant interaction between grammaticality and attractor number, which is the statistical demonstration of the asymmetry (for discussion about the need of interactions to support claims about asymmetric effects, see Gelman & Stern, 2006; Nieuwenhuis et al., 2011).

#### Second Issue: Attraction in Grammatical Sentences.

A second issue is that even when attraction is observed in grammatical sentences, its direction is unclear. Consistent with Pearlmutter et al. (1999), some studies have reported processing difficulty, consistent with representational accounts (Acuña-Fariña et al., 2014; Nicol et al., 1997; Patson & Husband, 2016). But many studies have found the opposite: processing facilitation (Franck et al., 2015; Nicenboim et al., 2018; Smith et al., 2021: experiment 1; Villata et al., 2018).

For example, Villata and colleagues (2018) conducted a self-paced reading study with 130 English participants. Their main analysis failed to show attraction effects online, but a supplementary analysis that included more trials (containing reading times up to 8,000 ms) found a marginal facilitatory attraction effect after the verb in grammatical sentences with plural attractors. These findings align with those of Nicenboim et al. (2018), who observed processing facilitation in grammatical sentences with plural attractors: in a sample of 184 German participants, the mean size of the effect at the verb was 9 ms and the range of effect sizes deemed likely with 95% probability was [0, 18] ms—this is known as a 95% Bayesian credible interval. This modest effect size is consistent with the estimate from a meta-analysis by Jäger et al. (2017): 13 [2, 28] ms. Thus, large participant samples seem necessary to detect attraction in grammatical sentences, although this is still uncommon in most studies on agreement. Our large-sample study seeks to address this limitation.

#### Third Issue: Encoding Interference.

In recent years, several researchers have suggested that reading time effects previously attributed to the retrieval triggered by a verb may instead be due to a different cognitive process: encoding interference (Ness & Meltzer-Asscher, 2017; Smith et al., 2021; Villata et al., 2018; Villata & Franck, 2020). Encoding interference arises when two elements share similar features, which degrades their distinctiveness in memory (Oberauer & Kliegl, 2006). Encoding interference reflects difficulty in the initial memory encoding of items, and thus it occurs prior to their retrieval by elements appearing later in a sentence, for example, verbs. Therefore, encoding interference effects should be observable prior to the appearance of a verb (in number attraction studies) or in configurations in which the manipulated feature(s) is not a retrieval cue.

The evidence for these two predictions is scarce and inconsistent in the attraction literature (Jäger et al., 2015; Parker & Konrad, 2020), but it is widespread in other grammatical domains.^1^ For instance, Gordon and colleagues (2002) reported that the comprehension of object relative clauses was facilitated when the subject and the object were of different types, for example, a definite description and a pronoun vs. two definite descriptions: “*The barber that **you*** / ***the lawyer** admired*.” The facilitation due to the pronoun was observed both at the second noun phrase and at the relative clause verb. While the effect at the pronoun itself could be due to lexical variables (pronouns are shorter and more frequent than definite descriptions), the reading times at the verb more strongly suggest an encoding effect, as the distinction between noun types is unlikely to be a retrieval cue (for similar findings see Barker et al., 2001; Fedorenko et al., 2006; Gordon et al., 2002; Hofmeister & Vasishth, 2014; Jäger et al., 2015; Kush et al., 2015).

In agreement attraction studies, some evidence of encoding interference has been provided by Villata et al. (2018). In addition to the experiment reported above, the authors conducted a second self-paced reading experiment that manipulated the gender (instead of the number) of attractor and target nouns in Italian object RCs, for example, “*The ballerina_FEM_ [that **the waiter***_***MASC***_ / ***the waitress***_***FEM***_ *has surprised]* … .” Results from 167 Italian participants showed that the participle verb was read more quickly when the attractor and target noun bore different gender, as opposed to when they matched. The surprising aspect of this result is that past participles in Italian object RCs do not instantiate gender agreement. Therefore, gender could not have possibly been used as a retrieval cue at the participle, disqualifying a retrieval-based explanation and favoring an encoding interference-based one.

Encoding interference effects in attraction cannot be explained by marking and morphing or by retrieval-based accounts. But they can be explained by a different framework, self-organized sentence processing (SOSP), which is the third account relevant for the present study (Kempen & Vosse, 1989; Smith et al., 2018). As the dynamics of SOSP are quite complex, here we offer only a brief summary focused on the two issues under discussion: attraction effects in grammatical sentences and the grammaticality asymmetry (for a detailed account, see Smith et al., 2021). In SOSP, encountering each word activates a treelet with semantic and syntactic features, similar to memory chunks in cue-based retrieval (e.g., +NP, −plural). Activated treelets attempt to combine in all possible ways with other activated treelets, and the strength of these connections grows over time. Crucially, and in contrast with cue-based retrieval, these interactions happen continuously, rather than only at retrieval. This allows SOSP to capture encoding interference effects.

For example, in the preamble “*the key to the cabinets*,” after the singular head noun “*key*” is encountered, the subject node of the sentence initially gravitates toward [+NP, −plural] (note that gravitation is a gradual process in which features increase their values from 0 toward a maximum of 1; for a useful visualization see Villata et al., 2018, figure 3). When the attractor is encountered later, its treelet competes with the “key” treelet to attach to the subject node. If the attractor is plural, competition is weak, because the attractor solely matches the subject node in the +NP feature. But if the attractor is singular, it will match both features of the subject node [+NP, −plural], increasing its competition with the head noun for attachment and delaying processing both prior and during the processing of the verb. The same competition mechanism operates in ungrammatical sentences, with the only difference that in this case the subject node is never a perfect match for the verb, regardless of which treelet attaches to it. Thus, SOSP predicts facilitation due to a plural attractor in both grammatical and ungrammatical sentences and it explains encoding and retrieval interference through the same mechanism.

The feature-competition property of SOSP also allows it to explain the grammaticality asymmetry. In grammatical sentences, all constraints align to form the correct parse, such that the attractor only has a weak influence. By contrast, in ungrammatical sentences, it is impossible to satisfy all the constraints and the system remains in a blended, intermediate state, with a benefit for the plural attractor condition due to reduced competition in the encoding of the subject phrase. In short, SOSP predicts processing facilitation in both grammatical and ungrammatical sentences as well as the grammaticality asymmetry, similarly to retrieval-based accounts and in contrast with the marking and morphing model. But in contrast to cue-based retrieval, SOSP can model encoding interference and explain attraction effects prior to the verb. However, studies to date have failed to find preverbal encoding interference effects, which is unexpected for SOSP. One potential explanation is that regions prior to the verb are seldom analyzed in reading studies (and when they are, the results are confounded by the lexical effect of attractor plurality, as shown by Wagers et al., 2009).

#### Fourth Issue: Differences Between Languages and Reading Paradigms.

A final problem in evaluating previous results lies in the scarcity and marked differences between studies. The review by Hammerly et al. (2019) shows that only six languages have been tested to date, with English comprising almost 90% of the evidence. Previous studies also range from self-paced reading and acceptability judgments to eye-tracking and event-related potentials. Considering that an important issue is the timing of verb-driven attraction effects vs. attractor-driven plurality effects, it is unfortunate that few studies have used the eye-tracking method (Dillon et al., 2013; Jäger et al., 2020; Parker & Phillips, 2017; Pearlmutter et al., 1999). Of these, the only non-English study was by Acuña-Fariña et al. (2014). Due to the speed and highly automatic nature of eye-movements, eye-tracking is a useful tool for examining temporal profiles and it also helps alleviate some concerns about response bias and task strategies that apply to judgment and self-paced reading tasks (Hammerly et al., 2019; Villata & Franck, 2020).

### The Current Study

Our work seeks to clarify the empirical status of attraction effects in comprehension. We focus on number attraction in Spanish, a language in which previous results are mixed and based on only two studies. Acuña-Fariña and colleagues (2014) used eye-tracking to measure the processing of prepositional phrase constructions in grammatical sentences such as “*The name of **the boy(s)** was German*” (attractor bolded). The results were similar to those of Pearlmutter et al. (1999), but attraction appeared immediately at the verb, which elicited more regressions and longer regression-path and total reading times when following a plural attractor. “Regression-path time” describes the reading times to a word prior to it being exited to the right, including the rereading of prior words.

In a later study, Lago and colleagues (2015) conducted four self-paced reading experiments that examined attraction effects in grammatical and ungrammatical sentences. They used object relative clauses such as “**The note(s)** [_RC_ that the girl writes/*write in the class] … .” As mentioned, object RCs avoid the confounding effects of plural morphology, because the plural attractor doesn’t immediately precede the verb and thus it is less likely to influence its reading. All experiments showed processing facilitation after ungrammatical verbs. However, the results in grammatical sentences were inconsistent: while no attraction was seen in one experiment, which used thematic verbs such as “*write*,” processing difficulty was observed with auxiliary verbs (“*is* *going to write*”): grammatical sentences with a plural attractor elicited longer reading times than those with singular attractors, consistent with representational accounts and with Acuña-Fariña et al. (2014). An additional experiment was performed to follow up on this effect, but it failed to replicate the attraction effect in grammatical sentences.

Taken together, these findings offer conflicting answers about the existence and directionality of attraction effects in Spanish grammatical sentences. On the one hand, the attraction effect in the study of Acuña-Fariña et al. (2014) may be attributed to the spillover effect of the attractor plurality, which may have confounded reading times at the verb. On the other hand, Lago et al. (2015) may have failed to observe this effect due to the limited temporal resolution of the self-paced reading method, which does not allow participants to reread prior material. This precludes the measurement of regressive eye movements and rereading patterns, two key markers of attraction in eye-tracking studies.

To address these issues, we revisited the grammaticality asymmetry in Spanish and sought to equalize testing conditions by using the same language, the same methodology, and the same design and linguistic constructions. We also recruited the largest number of participants that was feasible given our resources, motivated by previous findings that attraction effects in grammatical sentences are small and may require large samples to be detectable. We used the non-intervening configurations of Lago et al. (2015) to minimize spillover effects due to attractor plurality, but we employed an eye-tracking paradigm like Acuña-Fariña et al. (2014) to maximize the temporal resolution of our measures. We reasoned that eye-tracking should allow us to more comprehensively test for attraction in grammatical sentences, while replicating facilitation effects in ungrammatical ones. Further, the use of non-intervening configurations allowed us to compare reading profiles at the region before the critical verb, the RC subject region.

## EYE-TRACKING EXPERIMENT

Our study was a large-sample eye-tracking replication of the first experiment of Lago et al. (2015). The materials featured grammatical and ungrammatical sentences with a non-intervening attractor noun (e.g., “*the notes*”). An example is shown in (3) with the regions of analysis marked by vertical bars. These regions comprised the relative clause verb and a following 2–3-word adverbial (e.g., “*wrote in the class*,” henceforth, *verb*/*spillover region*). The region preceding the verb, consisting of the RC subject, was also analyzed to probe for encoding interference effects (e.g., “*la chica*,” *RC subject region*). This region was identical across conditions, thus avoiding lexical differences between conditions due to noun plurality (Wagers et al., 2009).

(3)  a. Grammatical, singular attractor
      **La nota** que | la chica | escribió | en la clase | alegró a su amiga.
     b. Grammatical, plural attractor
      **Las notas** que | la chica | escribió | en la clase | alegraron a su amiga.
     c. Ungrammatical, singular attractor
      *La nota que | la chica | escribieron | en la clase | alegró a su amiga.
     d. Ungrammatical, plural attractor
      *Las notas que | la chica | escribieron | en la clase | alegraron a su amiga.
     *Translation: The note(s) that the girl wrote*._*SG*_ / *wrote._*PL*_ *in the class cheered up her friend.*

Based on the attraction literature, we expected plural attractors to ease the processing of ungrammatical verbs, as reported by Lago et al. (2015) in Spanish and as predicted by SOSP, and representational and retrieval-based accounts. Our goal was to properly assess the magnitude of attraction effects in reading using a large participant sample and restricting our analysis to those eye-tracking measures independently supported by previous studies (see Analysis).

In addition, we sought to clarify the empirical record regarding the grammaticality asymmetry and the direction of attraction effects in grammatical sentences. If the grammaticality asymmetry holds in reading comprehension, we should obtain a statistical interaction between the number of the attractor noun and the grammaticality of the sentence (Wagers et al., 2009). Meanwhile, attraction in grammatical sentences is critical to arbitrate between different accounts. According to representational accounts, the plural attractor should disrupt the reading of the grammatical verb in (3b). According to retrieval-based accounts and SOSP, attraction should facilitate it. We note that due to the different perspectives adopted by these accounts, attraction effects *in grammatical sentences* are often reported inconsistently in the literature. Studies within representational and SOSP approaches treat the plural attractor condition as the condition of interest and compare it against the singular condition (which functions as a baseline condition). By contrast, retrieval-based studies treat the plural attractor condition as a baseline because their focus is on the singular attractor condition, in which similarity-based interference is expected in grammatical sentences. To avoid ambiguity, here we consistently take the plural attractor condition as the condition of interest in both grammatical and ungrammatical sentences, always using the singular attractor conditions as baseline (Dillon et al., 2013; Hammerly et al., 2019; Villata et al., 2018).

Finally, we assessed whether encoding interference contribute to attraction effects. The ability to explain encoding interference separates SOSP from representational and cue-based retrieval accounts. According to encoding interference one should be able to measure processing differences due to the attractor number prior to the verb. However, such effects have not been consistently found in online attraction studies, despite being attested in offline/untimed comprehension measures (Jäger et al., 2015; Villata et al., 2018) and in the comprehension of other linguistic phenomena (e.g., Gordon et al., 2004; Kush et al., 2015). Our study was optimized to detect encoding interference because the preverbal region was identical across conditions (thus avoiding a confound with noun plurality) and because the eye-tracking paradigm allowed measuring not only fixations but also regressive eye movements, a key diagnostic of attraction in previous studies.

### Methods

#### Participants.

Eye-movement data from 160 native speakers of Spanish were collected at the University of La Laguna in Tenerife, Spain. All participants had normal or corrected-to-normal vision. Due to experimenter error, their chronological age was not stored, but all participants were university students. Participants provided informed consent and received course credit or payment for their participation. All procedures were in accordance with the Declaration of Helsinki.

#### Materials.

Materials were taken from Lago et al. (2015). Some of them were modified in order to avoid uncommon expressions in Castilian Spanish like “lapicera” (‘pencil’), “aplicante” (‘applicant’), “reporte” (‘report’), “manejó” (‘drove’), and “fallas” (‘faults’), as well as Latin American geographical or cultural references, such as ‘the war of Paraguay,’ ‘the ancient Mayas,’ or ‘the Chilean border.’ The sentences were shortened to 80 characters when necessary to make them fit in one line of the display monitor.

The experimental sentences consisted of 48 sentence sets arranged in a 2 × 2 within-subjects design, with *grammaticality* (grammatical/ungrammatical) and *attractor number* (singular/plural) as factors. In the grammatical conditions, the relative clause (RC) subject and verb were both singular (i.e., they agreed in number), while in the ungrammatical conditions the RC subject was singular but the verb was plural. The RC verb was always in the simple past tense and perfective aspect. The singular suffix for this tense-aspect combination is one character long (e.g., “escribi-ó,” ‘write.3sg’), while the plural suffix is 4 characters long (e.g., “escribi-eron,” ‘write.3pl’). The attractor noun was always inanimate while the RC subject head was always animate.

The 48 sentence sets were distributed across four lists in a Latin square design, and were combined with 104 sentences from a different experiment (not reported here). These filler sentences were all grammatical and consisted of simple subject-verb-object-adverbial sentences like “The priest found the bishop in the morning.” This resulted in 15.8% of the items being ungrammatical. Materials, data, and analysis code are available at https://osf.io/f8bt7/.

#### Procedure.

Eye movements were recorded by a video-based Eyelink 1000 Plus sampling at 1000 Hz (SR Research). Sentences were presented in lowercase on a monitor that displayed up to 80 characters per line. When necessary, line breaks occurred after the critical region. Participants were seated 73 cm away from the monitor, and three characters equaled 1 degree of visual angle. Viewing was binocular but only the right eye was recorded.

Experimental and filler items were followed by yes/no comprehension questions to ensure that participants were attentive. In the experimental items, the questions never referred to the agreement dependency to avoid focusing participants’ attention on it. Thus, response accuracy was not a measure of interest in the experimental items. Each session began with a calibration on a 9-point grid and recalibration between trials was conducted if necessary. Participants were instructed to read at a natural pace and answer the comprehension questions. They were not informed that some sentences would contain grammatical errors. Three practice items were presented. Before each trial, participants fixated on a marker above the first word of the upcoming sentence. Upon fixation on this marker, the text appeared. The order of experimental and filler items was randomized for each list. An entire experimental session lasted approximately 30–45 min.

#### Analysis.

Eye-tracking data were processed as follows. Fixations equal or shorter than 80 ms within one character of another fixation were merged. We also merged fixations shorter than 40 ms falling within three characters of another fixation. All remaining fixations below 80 ms or above 800 ms were removed. Trials in which a region was skipped in initial reading were treated as missing data for that region.

Our analyses focused only on measures consistently found to show attraction in previous studies, to minimize the number of multiple comparisons, which can increase Type I error rates in eye-tracking (Godfroid & Hui, 2020; von der Malsburg & Angele, 2017). Within frequentists frameworks, this issue can be addressed by applying corrections, such as the Bonferroni correction (Bonferroni, 1936). This is not possible in a Bayesian framework—the framework used here—because the Bayesian approach is concerned with quantifying the strength of evidence for a hypothesis given its prior probability, regardless of the number of hypotheses under investigation (for discussion see Dienes, 2011). From a frequentist perspective, this may render the results anticonservative and, thus, they should be interpreted with caution.

Based on previous studies, we analyzed the probability of first-pass regressions (Dillon et al., 2013; Jäger et al., 2020; Pearlmutter et al., 1999), regression-path (Acuña-Fariña et al., 2014; Parker & Phillips, 2017), and total reading time (Acuña-Fariña et al., 2014; Dillon et al., 2013; Jäger et al., 2020; Parker & Phillips, 2017; Pearlmutter et al., 1999). The probability of first-pass regressions (also known as “regressions out”) is an early processing measure denoting the probability of initiating a regression when first encountering a region. By contrast, total time is a global measure that denotes the whole amount of time spent in a region, including rereading. Finally, regression-path time (also called “cumulative reading time” or “go-past time”) describes the amount of time spent since first entering a region from the left until leaving it to the right (including regressions to preceding regions).

Our critical region comprised the verb and spillover region, as shown in example (3). Following Cunnings and Sturt (2018), we analyzed these regions jointly and included Region as a fixed effect. This procedure helps minimize the number of statistical tests per region, it allows evaluating potential timecourse differences between regions and it increases power to observe small effects that may be nonsignificant at individual regions but nevertheless consistent across them. However, one drawback of the procedure is that pooling the regions in the same model assumes equal variances in both regions. This is unlikely to be true in our materials, in which the verb region comprised a single word, but the spillover region comprised a 2–3-word adverbial. Thus, we conducted an additional analysis of both regions separately. The results of this analysis were largely similar to those of the joint analysis, with the main difference concerning the first-pass regression measure (see the Supplemental Materials).

In addition to the *verb*/*spillover region*, we examined the reading of the relative clause subject, which appeared immediately before the verb: the *RC subject region*. If encoding interference occurs, the effect of the attractor number may already show up in the reading of the RC subject, prior to the appearance of the verb. Accordingly, previous eye-tracking studies have shown that the difficulty elicited by object RCs (in comparison to subject RCs) arises at the noun phrase following the relative pronoun (Staub, 2010b; Staub et al., 2017). For instance, Staub (2010b) showed that this noun phrase elicits more regressions and longer regression-path times than the same phrase in a subject RC or in a complement clause. Therefore, we examined first-pass regressions, regression-path, and total times in the RC subject region. We report this analysis in a separate section, Exploratory Analysis, because our decision to perform it was taken during the analysis stage, after data collection took place.

Reading data were analyzed with mixed-effects logistic regression (first-pass regression measure) and mixed-effects linear regression (regression-path and total time measures). All models were fit in a Bayesian framework using the brms package in R (Bürkner, 2017; R Development Core Team, 2020). Bayesian models are valuable because they combine prior information with evidence from the data in order to obtain a probability distribution over the plausible values of a parameter—the parameter’s posterior distribution. Thus, an experimental effect can be quantified in terms of the likelihood of its possible values, which is more informative than a binary statement about whether the effect is significant, because it puts the focus on determining an effect size and direction, along with its uncertainty (Cumming, 2014; Kruschke & Liddell, 2018).

The procedure for fitting Bayesian models and assessing their convergence followed Jäger et al. (2020). We used a hierarchical lognormal likelihood function to model the raw reading times in milliseconds. This is equivalent to log-transforming the values and fitting a hierarchical linear model with a normal likelihood. All models assumed correlated varying intercepts and slopes for items and participants for all predictors of interest and their interactions (Grammaticality, Attractor number, and Region; see Barr et al., 2013). Further, since there were two non-independent datapoints from each trial (e.g., one observation from the verb and one from the spillover region), we included a random intercept for trial, defined as each unique participant-item pairing.^2^

Two models were computed to address our research questions. The first model used fixed effects of Grammaticality, Attractor number, and Region, as well as their interaction. Its goal was to examine the existence of attraction and of the grammaticality asymmetry, which should appear as an interaction between grammaticality and attractor number. Since ungrammatical verbs were systematically longer than grammatical verbs, the factor region length was added to the models of the verb/spillover region (centered and operationalized as the number of characters per region).

The second model assessed the nested effects of attraction. Its goal was to estimate the effect of attraction in grammatical and ungrammatical sentences separately. Nested models were run for the three reading measures in the combined verb/spillover region, and for total times in the RC subject region. Separate nested models for grammatical and ungrammatical sentences were not run for first-pass regressions and regression-path times at the RC subject because these measures index processing prior to the appearance of the verb, when all conditions were grammatical. By contrast, total reading times include the second-pass reading of the RC subject region after it was exited to the right, and thus they may be affected by the grammaticality of the verb. In the nested model of the verb/spillover, Region was always used as a factor. In the nested model of the RC subject, which comprised a single identical region across condition, neither Region nor region length were used.

To avoid making strong a priori assumptions about possible effect sizes, we used mildly informative priors (Gelman et al., 2014). Specifically, we used a standard normal distribution *N* (0, 1) for all fixed effects except the intercept, which had an *N* (0, 10) prior. The prior for the random effects and the residual variance used a half-normal distribution *N*^+^ (0, 1), because random effects and residual variance cannot be negative. Within the variance-covariance matrices of the by-participant and by-item random effects, priors were defined for the correlation matrices using a so-called Lewandowski-Kurowicka-Joe (LKJ) prior (Lewandowski et al., 2009). This prior has a parameter η, which, when set to 2, has the regularizing effect of disfavoring extreme correlations.

All contrasts were sum-coded as ± 0.5, such that the model parameters reflected differences between condition means. For the factor grammaticality (−0.5 grammatical / +0.5 ungrammatical), a positive coefficient reflects a slowdown in ungrammatical sentences, that is, processing disruption. For attractor number (−0.5 singular / +0.5 plural), a negative coefficient reflects a speedup for sentences with a plural attractor. For the grammaticality × attractor number interaction, a negative coefficient shows stronger attraction effects in ungrammatical than grammatical sentences. For the factor region (−0.5 verb / +0.5 spillover), a positive coefficient reflects longer reading times (or more regressions) in the spillover than in the verb region.

### Results

Mean accuracy in the comprehension questions was 91.4% (*SD* = 28%). For the reading data, we focus on the effects of interest but include the full output of the main models in the Supplemental Materials. The Supplemental Materials also contain the mean reading times in all sentence regions, to provide a visual summary of how readers navigated the sentences (Figure S1). We report the mean of the posterior probability distribution of each effect together with a 95% credible interval (CrI), which represents the interval where it is 95% certain that the true effect lies given the data and the model. For easier interpretability, reading measures are back-transformed to their original scale in the text, but statistical analyses and inferences are based on the untransformed model coefficients.

#### Verb and Spillover Region.

**Figure 1.**
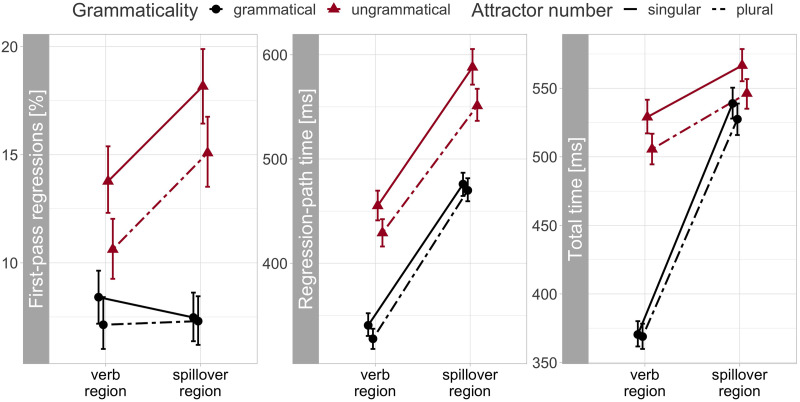
**Empirical reading measures for the verb (“wrote._*SG*/*PL*_”) and spillover region (“in the class”).** Points show condition means and error bars 95% confidence intervals calculated for each condition across participants and items.

**Figure 2.**
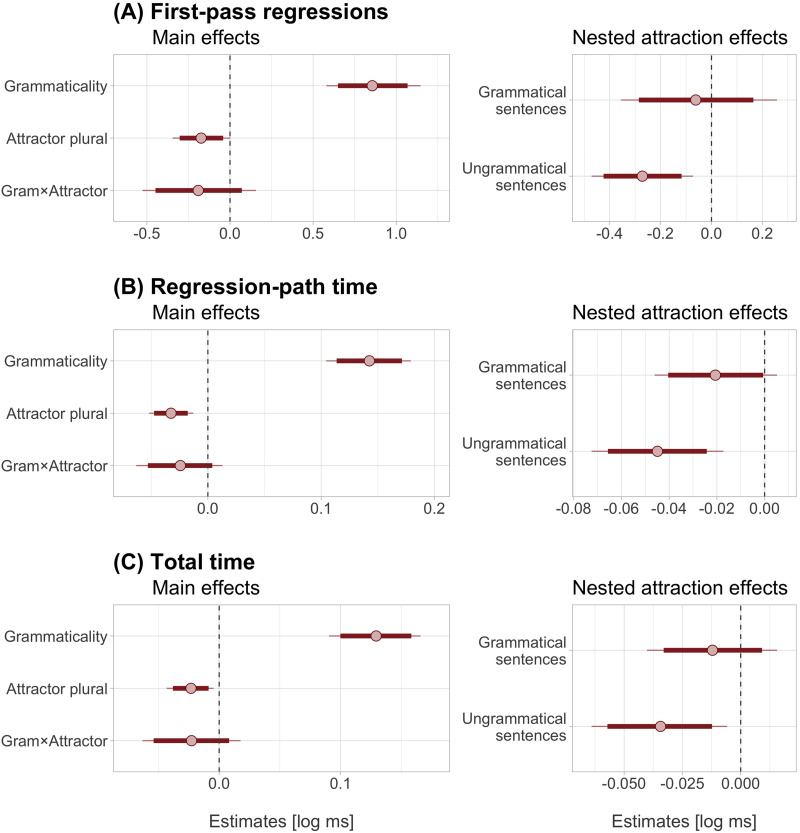
**Posterior estimates of the statistical model for first-pass regressions, regression-path, and total time measures in the verb/spillover region.** Red circles show posterior means and thicker horizontal lines show 95% credible intervals (thinner lines show 99% credible intervals). Dashed vertical lines correspond to an effect size of zero. For the factor grammaticality, positive estimates reflect longer reading times (or more regressions) in ungrammatical sentences. For the attractor factor, negative estimates reflect faster reading times (or fewer regressions) in plural attractor sentences. For the interaction, negative estimates suggest stronger attraction effects in ungrammatical than grammatical sentences.

The verb region was skipped on 2.7% of trials and the spillover region on 0.56% of trials. The reading patterns were consistent across measures (Figure 1). All measures showed main effects of grammaticality and attraction (Figure 2). Specifically, ungrammatical verbs disrupted processing by eliciting more regressions (posterior mean 6.1%, 95% CrI [4.5, 7.7] %) and longer regression-path (55 [44, 67] ms) and total times (56 [43, 69] ms). Meanwhile, plural attractors facilitated processing by reducing regressions (−1.2 [−2.2, −0.3] %), regression-path (−13 [−18, −7] ms), and total times (−10 [−17, −4] ms). Finally, the mean estimates of the grammaticality × attractor interaction were negative across measures, consistent with larger attraction effects in ungrammatical than grammatical sentences. However, although the credible intervals were clustered on negative values, they also included zero and a few positive values: −1.3 [−3.2, 0.5] % regressions, −9 [−21, 1] ms regression-path, and −10 [−23, 4] ms total time. Thus, these results are consistent with the claim of larger attraction effects in ungrammatical sentences—the grammaticality asymmetry—but they do not conclusively support it.

The nested model assessed the size of attraction effects separately for ungrammatical and grammatical verbs. Ungrammatical verbs showed facilitation due to attraction: verbs preceded by plural attractors elicited fewer regressions (−1.9 [−3.1, −0.7] %) and faster regression-path (−17 [−25, −9] ms) and total times (−15 [−25, −6] ms) than verbs preceded by singular attractors. Grammatical verbs showed similar facilitatory patterns, but attraction effects were inconclusive because they spanned negative and positive values: −0.5 [−2, 0.9] % regressions, −8 [−16, 0] ms regression-path and −5 [−14, 4] ms total time.

#### Exploratory Analysis: RC Subject Region.

**Figure 3.**
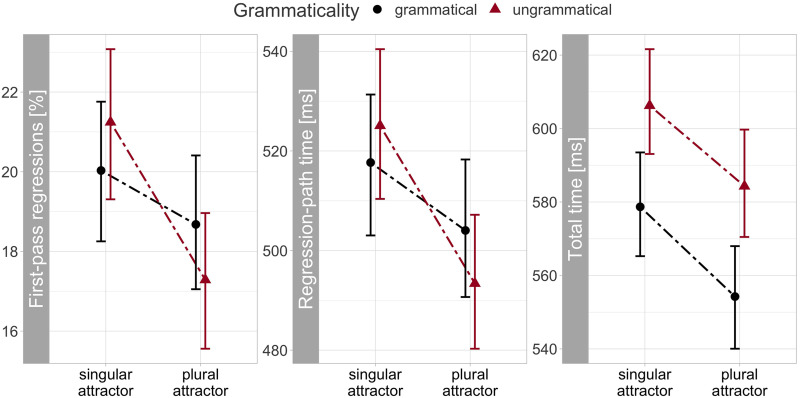
**Empirical reading measures for the relative clause subject (“the girl”).** Points show condition means and error bars 95% confidence intervals calculated for each condition across participants and items.

The RC subject was skipped on average on 2.27% of trials. The earlier processing measures, first-pass regression and regression-path times, did not show a grammaticality effect, as expected since all conditions were grammatical prior to the verb. But the number of the attractor noun modulated regression-path times: when the attractor was plural, the RC subject—which was always singular—elicited shorter regression-path times: −17 [−29, −5] ms. Thus, reading was facilitated when the attractor and the RC subject bore different numbers, consistent with encoding interference (Figure 3). A similar pattern was observed in first-pass regressions, with fewer regressions after a plural attractor: −2.7 [−4.8, −0.8] %. However, this effect should be taken with caution, because visual inspection of Figure S1 (Supplemental Materials) revealed a similar pattern already in the preceding region. Therefore, we can’t rule out that the encoding effect at the RC subject in first-pass regressions was due to spillover from the previous region.

**Figure 4.**
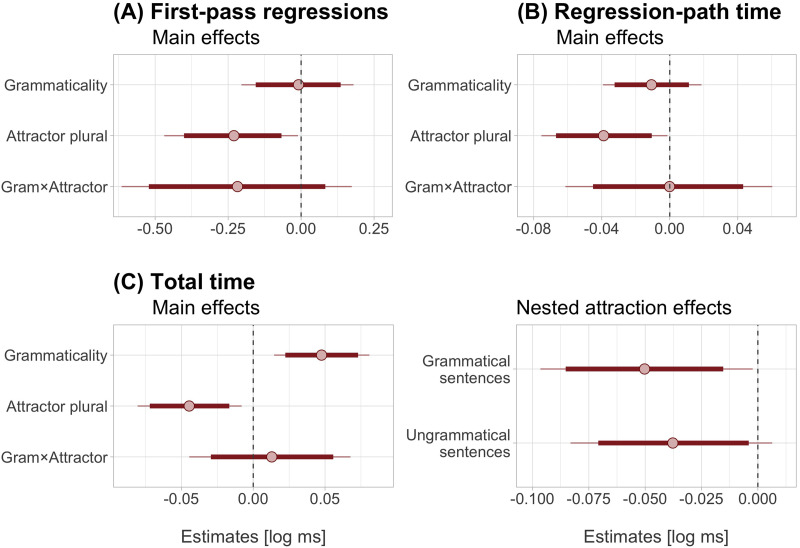
**Posterior estimates of the statistical model for first-pass regressions, regression-path, and total time measures in the verb/spillover region.** Red circles show posterior means and thicker horizontal lines show 95% credible intervals (thinner lines show 99% credible intervals). Dashed vertical lines correspond to an effect size of zero. For the factor grammaticality, positive estimates reflect longer reading times (or more regressions) in ungrammatical sentences. For the attractor factor, negative estimates reflect faster reading times (or fewer regressions) in plural attractor sentences. For the interaction, negative estimates suggest stronger attraction effects in ungrammatical than grammatical sentences.

The RC subject showed a clear effect of grammaticality in total reading times (24 [11, 37] ms) and also of the number of the attractor noun, with shorter total reading times when the attractor and the target noun bore different numbers: −22 [−36, −8] ms. The nested comparisons showed that the number mismatch between the attractor and target noun facilitated processing in ungrammatical and grammatical sentences similarly: −19 [−36, −2] ms and −26 [−43, −8] ms, respectively (Figure 4).

## GENERAL DISCUSSION

Our study examined the reading profiles of subject-verb agreement attraction in Spanish. We were interested in whether attraction effects would differentially disrupt the comprehension of grammatical and ungrammatical sentences, giving rise to the so-called grammaticality asymmetry. In Spanish, to our knowledge, only two studies have examined the reading profiles of number attraction, and only one of them probed for the grammaticality asymmetry. But their direct comparison is challenging because they used different designs, grammatical constructions, and methods. Our study sought to equalize testing conditions by deploying the same design and linguistic constructions as Lago et al. (2015) but using eye-tracking and a large participant sample to increase the sensitivity of the measurements.

Our results can be summarized as follows. First, we replicated the finding that attraction eases the reading of ungrammatical verbs, as predicted by all the accounts of attraction considered here: SOSP, representational, and retrieval-based accounts. Facilitatory effects were observed in all reading measures: first-pass regressions, reading-path, and total times. These attraction effects were small. For comparison, the disruption due to an ungrammatical verb (as opposed to a grammatical one) increased regression-path and total reading times 50 ms on average and the regression probability about 6%. Meanwhile, attraction-induced facilitation in ungrammatical sentences was on average 13 ms in reading-path times, 10 ms in total times, and less than 2% in first-pass regressions. These modest sizes are not unexpected: Large sample studies can provide more realistic estimates of an effect size, as significant effects in low-powered studies are often overestimates (Vasishth et al., 2018).

Our remaining results concern the empirical effects with a mixed record in previous studies: the grammaticality asymmetry and attraction effects in grammatical sentences. The direction of attraction effects in grammatical sentences is crucial to arbitrate between processing models, because both SOSP and retrieval-based predict facilitation with grammatical verbs, while representational accounts predict processing difficulty. Unfortunately, our analyses failed to detect conclusive effects of attraction in grammatical sentences in the verb/spillover region. However, the mean estimates of the effects, as well as their credible intervals, consistently suggested facilitation, with faster reading times and fewer regressions in the plural attractor condition.

Based on these reading profiles, our view is that our results align with those of large-sample studies and of a meta-analysis (Jäger et al., 2017; Nicenboim et al., 2018; Villata et al., 2018) and suggest that attraction in grammatical sentences is unlikely to emerge as processing difficulty during reading. Under this view, the unexpected results in previous studies are those that showed processing difficulty in grammatical sentences (Acuña-Fariña et al., 2014; Nicol et al., 1997; Pearlmutter et al., 1999; Patson & Husband, 2016). Since all these studies used prepositional phrase constructions like “*The key to the **cabinet(s)** is* …,” a likely explanation is that the spillover effects of noun plurality created the processing difficulty measured at the grammatical verb (Wagers et al., 2009). We mitigated this confound by testing object RCs, in which the plural attractor did not immediately precede the verb. We believe that object RCs provide a better way to diagnose attraction in grammatical sentences, but we acknowledge an alternative proposal that attraction effects in relative clauses may obey a different mechanism, so-called “predication confusion” (Staub, 2009, 2010a). While we cannot rule out this possibility, under this proposal it is surprising that prepositional phrases and object RCs often show the same attraction effects. Experiments using other constructions (or languages in which modifiers linearly precede their heads) may help resolve this issue.

### Encoding Interference in Agreement Attraction

The novel finding from our study is that participants read the RC subject more easily when it differed in number with the preceding attractor noun, and that this effect emerged prior to the critical verb. In the plural attractor condition, regression-path times for the singular RC subject were reduced on average 17 ms and regressions decreased about 3%. Total reading times, which incorporate refixations to the RC subject after encountering the verb, showed equivalent reductions in ungrammatical and grammatical sentences: on average 20 and 25 ms, respectively. Crucially, facilitation effects were similarly sized in grammatical and ungrammatical sentences at the RC subject, but they were numerically larger for ungrammatical sentences at the verb/spillover region. This suggests that the two effects are unlikely to reflect the same process. While the grammaticality asymmetry at the verb/spillover region is expected under retrieval-based accounts, the symmetric effect at the RC subject requires an additional explanation.

Regressions and regression-path time measures at the RC subject reflect processing prior to the verb, and so the effect at the RC subject cannot be explained by retrieval-based accounts. However, one explanation that would still be consistent with a retrieval-based account is that the effect at the RC subject reflected a parafoveal-on-foveal effect, such that the parafoveal processing of the RC verb was complete enough to trigger the retrieval of an object and affect fixations durations at the RC subject. We think that this is unlikely for two reasons. First, under such an explanation—without additional assumptions—we should have also found a grammaticality effect at the RC subject. Second, the existence of parafoveal-on-foveal effects in experimental reading paradigms is uncertain (Schotter, 2018). Most of the supporting evidence for these effects comes from corpus analyses, which lack tight experimental controls (Kennedy & Pynte, 2005; Kliegl et al., 2006). Meanwhile, experimentally controlled studies have found little to no evidence of parafoveal-on-foveal effects of the lexical properties of a following word, such as lexical frequency, predictability, and plausibility (Angele et al., 2008; Angele & Rayner, 2011; Angele et al., 2015; Henderson & Ferreira, 1993; Inhoff et al., 2000; Rayner et al., 2007; Staub et al., 2007). Thus, we think that parafoveal-on-foveal effects are an unlikely explanation for the reading patterns at the RC subject region.

Instead, the effect at the RC subject is consistent with encoding interference. Within memory-based frameworks, encoding interference can be conceptualized as a feature overwriting process (Nairne, 1990; Oberauer & Kliegl, 2006). The proposal is that shared features increase the competition between two items, reducing their distinctiveness and thus their memory activation. Encoding interference effects are common in psycholinguistics. For instance, the comprehension of object relative clauses is facilitated when the subject and object are of different types, for example, a definite description and a pronoun vs. two definite descriptions: *The barber that **you*** / ***the lawyer** admired* (Gordon et al., 2002; see also Barker et al., 2001; Fedorenko et al., 2006; Hofmeister & Vasishth, 2014). Similarly, it has been shown that participants read words within a cleft clause more slowly when they rhyme with those of a currently maintained memory word list, and that this slowdown occurs prior to the verb/retrieval site (Kush et al., 2015).

Surprisingly, encoding interference is seldom found in online attraction studies, despite being common in offline comprehension measures (Adani et al., 2010, 2014; Belletti et al., 2012; Jäger et al., 2015; Parker & Konrad, 2020; Villata & Franck, 2020). Only two previous self-paced reading studies reported effects consistent with encoding interference, but these effects were found only when including very long reading times (typically considered outliers in self-paced reading; Villata et al., 2018) or they were inconsistent across experiments (Smith et al., 2021). One explanation for why we were able to detect encoding interference is that we used an eye-tracking paradigm, which can capture difficulties in information encoding by measuring regressive eye movements. By contrast, self-paced reading studies don’t allow participants to reread previous material, and, thus, encoding difficulty can only emerge as excessively long reading times, which are likely to be discarded during analysis.

Of the theoretical accounts discussed here, only SOSP can capture encoding interference effects. This is because SOSP describes the building of structure as a competition between treelets with semantic and syntactic features, such that the treelets corresponding to the attractor and target noun compete to attach to the treelet corresponding to the RC subject node. When the attractor and target noun overlap in features, competition for attachment is stronger, which delays processing prior to the verb in grammatical and ungrammatical sentences alike.

By contrast, current retrieval-based accounts cannot explain encoding interference, in either their activation-based (Lewis & Vasishth, 2005) or direct access implementations (Nicenboim & Vasishth, 2018). This is because these accounts assume that interference is due to an overlap between the retrieval cues of the verb and the features of previously encoded items in memory. Therefore, attraction can only emerge after the verb is encountered, as the verb initiates the retrieval process. However, the dynamics of encoding interference are similar to that of another mechanism that is already part of the cue-based retrieval framework: the fan effect (Anderson et al., 2004; Lewis & Vasishth, 2005). The fan effect penalizes the retrieval of an item when the retrieval cue also matches other items, that is, when a feature is shared by two or more items. The critical difference is that the fan effect only operates at retrieval, while encoding interference affects the creation of items in memory (Jäger et al., 2015).

As pointed out by Villata et al. (2018), incorporating a fan-like effect at encoding in a cue-based retrieval framework creates new challenges. In current implementations, the activation level of a newly created memory chunk does not depend on its feature overlap with other chunks. This assumption would need to be changed to penalize items whose features overlap with those of previously encoded items (e.g., an “activation leveling” process, as suggested by Villata et al., 2018). Notwithstanding the specific computational implementation, incorporating a mechanism to generate encoding interference will be critical for future cue-based retrieval implementations, if they are to appropriately capture the processing patterns attested in the current study.

### Limitations and Open Questions

Our study has several limitations and it leaves some open questions. The first concerns our research question about the existence of a grammaticality asymmetry, that is, the possibility that attraction affects grammatical and ungrammatical Spanish sentences differentially. The statistical correlate of the grammaticality asymmetry is an interaction between grammaticality and attraction, but our interaction effects were not conclusive. This is puzzling, as all reading measures showed quantitatively larger attraction effects in ungrammatical than grammatical sentences. However, the posterior probability distributions of the interaction in Figure 2 also suggest a broad distribution for these effects, across a relatively large set of values. This indicates that an even more highly powered design might be needed to increase the precision of the estimates in order to reliably detect the interaction.^3^ The estimates obtained here could be used by future studies in Spanish to run power analyses in order to estimate the sample size needed to detect such evidence.

Second, previous work has proposed that the grammaticality asymmetry can be explained under a view of retrieval as a repair-based mechanism (Lago et al., 2015; Schlueter et al., 2019; Wagers et al., 2009). The hypothesis was that participants only used memory retrieval to “repair” a sentence after a number violation had already been encountered. This repair-based hypothesis predicts that reading disruptions due to ungrammaticality should temporally precede attraction effects, as readers should first detect an agreement violation and only afterward experience attraction due to misretrieval. In our study, we did not find evidence of an earlier onset of grammaticality vs. attraction effects. First-pass regressions, which index early processing, already showed evidence of both effects. Therefore, our results fail to support a repair-based view of retrieval. While such evidence might have emerged if other measures of early processing had been examined, we limited ourselves to measures that have shown attraction in previous studies, to reduce the number of multiple comparisons. Further, the use of other early processing measures would be unadvisable, since these are particularly sensitive to lexical properties like word length and frequency (Rayner, 1998). In our study, ungrammatical verbs were always plural and thus longer and less frequent than grammatical verbs. This problem is common in attraction studies (Avetisyan et al., 2020; Dillon et al., 2013; Lago et al., 2015; Schlueter et al., 2019). While we tried to minimize it by using region length as a predictor in the analysis, future studies might consider a different manipulation of grammaticality to better compare it with attraction effects.

Finally, our finding of encoding interference at the RC subject was only obtained in an exploratory analysis. This analysis was labeled “exploratory” because, even though encoding effects are consistent with the SOSP account of attraction, we only thought of performing it during the analysis stage, as we became acquainted with recent work on attraction in online comprehension (Villata et al., 2018; Smith et al., 2021). Therefore, it will be important for future studies to replicate our finding, especially due to its implication for theoretical accounts of attraction, either in terms of supporting an SOSP framework or in showing the need to incorporate a feature overwriting process in cue-based retrieval models. Crucially, our findings suggest that to detect encoding interference effects in comprehension, the use of large participant samples is advisable.

## ACKNOWLEDGMENTS

We thank Anna Laurinavichyute, Garrett Smith, and two anonymous reviewers for useful feedback and discussions.

## AUTHOR CONTRIBUTIONS

SL: Conceptualization: Equal; Data curation: Lead; Formal analysis: Supporting; Writing – original draft: Lead; Writing – review & editing: Lead. CAF: Conceptualization: Equal; Writing – original draft: Equal; Writing – review & editing: Equal. EM: Conceptualization: Equal; Data curation: Equal; Formal analysis: Equal; Investigation: Lead; Resources: Lead; Writing – original draft: Equal; Writing – review & editing: Equal.

## Notes

^1^ But note that number attraction effects that are consistent with encoding interference in grammatical sentences have been attested in question comprehension measures in adult and developmental studies in German, Swedish, Hebrew, and Italian (Adani et al., 2010, 2014; Belletti et al., 2012; Jäger et al., 2015; Villata & Franck, 2020). However, since attraction effects were only probed with offline measures, we do not discuss them in the main text because we focus on processing effects.^2^ We also tried to include a by-region slope for the trial random effect. But this led to several problems, including divergent transitions, an R-hat statistic higher than 1.01 and chains with a low estimated Bayesian Fraction of Missing Information. Because the problem persisted after changing the optimization parameters and doubling the number of iterations (Stan Development Team, 2020; https://mc-stan.org/misc/warnings.html), we removed the by-region slopes for the models reported in this article.^3^ Finding an interaction may have also been particularly challenging because attraction effects in our study had the same direction in grammatical and ungrammatical sentences. This may help explain why reports of the grammaticality asymmetry are inconsistent across studies. As mentioned in the Introduction, previous studies have coded comparisons in the grammatical sentences differently, some taking the plural attractor condition as a baseline and others doing the reverse. With the former coding, attraction effects go in opposite directions in grammatical and ungrammatical sentences, which maximizes the likelihood of finding a significant interaction. We do not have a solution to this problem, as the alternative coding schemes are motivated by different theoretical accounts. Here we decided to use a coding scheme that reflected the linguistic manipulation in our materials because it seemed like a more theory-neutral way to describe the data.

## Supplemental Materials

https://osf.io/f8bt7/
