# Erratum

**DOI:** 10.1162/opmi_e_00106

**Published:** 2023-10-01

In “The Reading Signatures of Agreement Attraction” by Lago, S., Acuña Fariña, C., & Meseguer, E. [*Open Mind: Discoveries in Cognitive Science*, *5*, 132–153, 2021. https://doi.org/10.1162/opmi_a_00047], the following sentence (p. 136) “This modest effect size is consistent with the estimate from a meta-analysis by Jäger et al. (2017): 13 [2, 28] ms.” should read: “This modest effect size is consistent with the estimate from a meta-analysis on attraction effects in agreement dependencies by Jäger et al. (2017), which indicates a small facilitatory effect: −7 [−16, 4] ms.”

